# Analysis of trigeminal nerve disorders after oral and maxillofacial intervention

**DOI:** 10.1186/1746-160X-6-24

**Published:** 2010-10-26

**Authors:** Sareh Said Yekta, Felix Koch, Maurice B Grosjean, Marcella Esteves-Oliveira, Jamal M Stein, Alireza Ghassemi, Dieter Riediger, Friedrich Lampert, Ralf Smeets

**Affiliations:** 1 Department of Conservative Dentistry, Periodontology and Preventive Dentistry, Aachen University, Germany; 2 Interdisciplinary Center for Clinical Research, Aachen University, Germany; 3 Oral and Maxillofacial Surgery, University medical centre of the Johannes Gutenberg University Mainz, Mainz, Germany; 4 University of Basel, Hightech Research Center (HFZ) of Cranio-Maxillofacial, Surgery, Basel, Switzerland; 5 Department of Oral and Maxillofacial Surgery, Aachen University, Germany

## Abstract

**Background:**

Quantitative sensory testing (QST) is applied to evaluate somatosensory nerve fiber function in the spinal system. This study uses QST in patients with sensory dysfunctions after oral and maxillofacial surgery.

**Methods:**

Orofacial sensory functions were investigated by psychophysical means in 60 volunteers (30 patients with sensory disturbances and 30 control subjects) in innervation areas of the infraorbital, mental and lingual nerves. The patients were tested 1 week, 4 weeks, 7 weeks and 10 weeks following oral and maxillofacial surgery.

**Results:**

QST monitored somatosensory deficits and recovery of trigeminal nerve functions in all patients. Significant differences (p < 0.05) between control group and patients were shown for cold, warm and mechanical detection thresholds and for cold, heat and mechanical pain thresholds. Additionally, QST monitored recovery of nerve functions in all patients.

**Conclusion:**

QST can be applied for non-invasive assessment of sensory nerve function (Aβ-, Aδ- and C-fiber) in the orofacial region and is useful in the diagnosis of trigeminal nerve disorders in patients.

## Background

Nerve injury-associated dysfunction is a frequently reappearing problem in dentistry. After Oral- and Maxillofacial Surgery, many patients suffer from paresthesia or sensory loss in the perioral region. Inferior alveolar nerve and lingual nerve injuries are the leading cause of litigation and patient complaints in the field of oral surgery [1] and often an expert's report with a precise evaluation of the severity is needed.

Unfortunately, full comprehension of the underlying pathophysiology as well as an appropriate treatment seems to be missing [2-4]. In clinical practice, diagnostic means are mostly limited to sharp-blunt discrimination both to diagnose sensory neuropathy and to examine its regeneration [5]. An accurate, mechanism based diagnosis, which contains a comprehensive characterization of the somatosensory phenotype of the patients, however, is of utmost importance to understand the underlying pathophysiological mechanisms of neurosensory disturbance [6]. There are qualified and non-invasive methods, e.g. recording of trigeminal somatosensory evoked potentials after stimulation of hairy skin or oral mucosa to quantify sensory dysfunction [7-13] or visualisation of brain activities by functional magnetic resonance imaging to assess sensory function [14,15], but these methods are complex and extensive.

Quantitative sensory testing (QST) is a reliable, non-invasive psychophysical test of large- and small-fiber sensory modalities [16], which has become an implementable diagnostic tool [17-20]. In order to afford comparable testing results, a standardized QST battery of 13 thermal and mechanical parameters has been developed [6].

This QST approach has already been used in the face [21], and normative data for extraoral and intraoral regions have been collected and calculated [22].

The present study utilized this standardized QST battery, adapted to the trigeminal region, to test the sensory function of patients in the mental, infraorbital or lingual region following different interventions in oral and maxillofacial surgery. Regeneration characteristics of the investigated afferent fibres were analysed and ways of reducing the extent of the testing battery without affecting the informative value of the measurement were looked for. The study also presents QST as a useful tool for expert's reports.

## Methods

Orofacial sensory functions were investigated by psychophysical means in 60 volunteers (30 patients and 30 sex- and age matched control subjects) covering an age range between 17 and 81 years (43.4 ± 19.4 years, mean ± standard deviation (SD)). Only patients who identified paresthesia postoperative were tested. Exclusion criteria were as follows: neurological or psychiatric history, diabetes, and medication within 48 h. All participants gave their informed consent prior to their inclusion in the study according to the 1964 Declaration of Helsinki (as amended by the 59th General Assembly, 2008; http://www.wma.net). The protocol was approved by the local ethics committee.

Thermal and mechanical detection and pain thresholds were determined by the quantitative sensory testing protocol (QST) that contained originally 13 parameters [6,21]: CDT, cold detection threshold; WDT, warm detection threshold; TSL, thermal sensory limen; PHS, paradoxical heat sensation; CPT, cold pain threshold; HPT, heat pain threshold; MDT, mechanical detection threshold; MPT, mechanical pain threshold; MPS, mechanical pain sensitivity; ALL, allodynia; WUR, wind-up ratio; VDT, vibration detection threshold; PPT, pressure pain threshold.

### Quantitative Sensory Testing, QST

Thermal stimuli were applied by a computer controlled Peltier type thermode with a stimulation area of 16 × 16 mm^2 ^(TSA-II, medoc Ltd., Israel). Starting from a baseline of 32°C, temperature decreased or increased by 1°C/s in order to determine CDT, WDT, CPT, and HPT. Volunteers were asked to press a computer mouse button as soon as they perceive the corresponding cold, warm, cold pain, or heat pain sensation. After indicating perception, temperature of the thermode returned back to baseline. The range of stimulation temperatures was between 0°C and 50°C. CDT and WDT were specified as difference temperatures (dT) from baseline (32°C), CPT and HPT were defined as absolute temperatures (°C) [22]. Additionally, TSL was determined by alternating warm and cold stimuli. From the 32°C baseline, temperature increased until the indication of warm perception by the subject caused a decrease of temperature down to a cold perception and vice versa. This alternating stimulus series was repeated three times from warm to cold perception and from cold to warm perception. The mean difference between temperatures causing warm and cold perceptions was defined as TSL. In the same test, possible paradoxical heat sensations (PHS, a subjective feeling of heat upon cooling) during cold stimuli were registered.

MDT was measured with modified von Frey filaments with forces of 0.08, 0.2, 0.4, 0.7, 1.6, 4, 6, 10, 14, 20, 40, 60, 80, 100, 150, 260, 600, 1000, 1800, 3000 mN, (Touch-Test Sensory Evaluators, North Coast Medical, CA, U.S.A.). Custom-made weighted pinprick stimulators with forces of 8, 16, 32, 64, 128, and 256 mN and a contact area of about 0.2 mm diameter were applied in order to measure MPT. MDT and MPT were determined by the method of limits starting with a clearly noticeable filament of 16 mN and a usually non painful pinprick stimulator of 8 mN, respectively [23]. MDT and MPT were defined as the geometric mean of five series of descending and ascending stimulus intensities. MPS and ALL were acquired by a series of 30 pinprick stimuli and 15 light tactile stimuli in a pseudo-randomized order. Six different pinprick stimuli (8 to 256 mN, see above) were applied five times each. Light tactile stimulations were performed by a cotton wisp (about 5 mN), a cotton wool tip fixed to an elastic strip (about 100 mN), and a brush (about 200 to 400 mN; SENSELabTM Brush 05, SOMEDIC, Sweden). These three light tactile stimuli were applied three times each (single stroke of 1 to 2 cm length) intermingled with pinpricks. Subjects were asked to rate sensory sensations on a numerical scale: 0 defined as "no pain", 1 to 100 defined as "painful", 100 defined as "maximum imaginable pain". Stimulus-response-functions for MPS were calculated as geometric means of individual ratings. The wind-up phenomenon was acquired by applying a single pinprick stimulus (128 mN, see above) and a subsequent series of 10 pinprick stimuli with an inter-stimulus interval of 1 sec within a skin area of about 1 cm^2^. The subjects gave one pain rating each for the single stimulus and for the complete 1 Hz stimulation series on a numerical rating scale (cf. MPS, see above). This procedure was performed five times. The mean pain rating of trains divided by the mean pain rating to single stimuli was calculated as WUR.

Vibration stimuli were applied by a 64 Hz Rydel-Seifer tuning fork (OF033N, Aesculap, Tuttlingen, Germany) that was placed over maxilla (infraorbital nerve area) or mandible (mental nerve area). Threshold measurement was performed three times on one side starting with maximum vibration amplitude. As soon as the subject indicated disappearance of vibratory sensation the threshold was read on a scale ranging from 0/8 to 8/8 (steps of 1/8). VDT was defined as the arithmetic mean of three runs.

PPT has to be conducted on the masticatory muscles with a force gage device (FDN 200, Wagner Instruments, U.S.A.). The stimulator had a circular probe of 1.1 cm diameter that exerted pressures uo to 2000 kPa. Pressure was increased with 50 kPa/s until deep muscle pain was evoked. PPT was defined as arithmetic mean of three stimuli.

Patients were tested in innervation areas of infraorbital nerve (hairy skin, upper lip) (10 patients), mental nerve (hairy skin, lower lip) (10 patients), and lingual nerve (glabrous skin, anterior lateral two-thirds of the tongue) (10 patients) 1 week, 4 weeks, 7 weeks and 10 weeks following different interventions in oral- and maxillofacial surgery (zygomatic fracture surgery, dysgnathia surgery, third molar surgery, apicoectomy, implant insertion).

In the first intervention 1 week after surgery, not only the operated side (test side), but also the contralateral side (control side) was tested. The contralateral side was tested first.

The control group underwent the same tests on both sides once.

QST data on both sides were obtained within one experimental session, which took ~ 1 h, including a demonstration of each test at a practice area. Subjects laid on a couch and kept their eyes closed throughout the QST procedure. All investigations were performed by the same trained examiner.

In infraorbital and mental regions 12 parameters were determined (CDT, WDT, TSL, PHS, CPT, HPT, MDT, MPT, MPS, ALL, WUR, VDT). As the measurement of PPT was painful for the patient in many cases, this parameter was omitted. On the tongue QST protocol was adapted to seven parameters: CDT, WDT, TSL, PHS, CPT, HPT, MDT.

Tests were conducted within the infraorbital nerve territory on hairy skin of upper lip, within the mental nerve territory on hairy skin of lower lip, and within the lingual nerve territory on the anterior lateral two-thirds of tongue mucosa.

For all thermal QST parameters Friedman Repeated Measures ANOVA (Chisquare = χ^2^, p value) and subsequent Student-Newman-Keuls test (q, p value) were performed. Correlations between quantitative sensory variables and age were analyzed by Pearson's correlation analysis. Level of significance was set to p < 0.05. Statistical analysis was performed by the Software SigmaStat 3.0 (SPSS Inc., U.S.A.).

## Results

60 participants were tested in innervation areas of infraorbital nerves (hairy skin, upper lip) (10 patients and 10 control subjects), mental nerves (hairy skin, lower lip) (10 patients and 10 control subjects), and lingual nerves (glabrous skin, tongue) (10 patients and 10 control subjects). The patients were tested 1 week, 4 weeks, 7 weeks and 10 weeks following different interventions in oral and maxillofacial surgery (zygomatic fracture surgery, dysgnathia surgery, third molar surgery, apicoectomy, implant insertion). One week after surgery, both control and test side were investigated in the patient group. The volunteers of the control group were tested once, in the same innervation area as their respective patient.

The values of the control group were all in normal range. The values of the control side (patient group) were all in normal range, too.

There were no significant differences between the values of the control group and the control side values of the patient group.

### Differences between control data and test data 1 week after surgery

**Figure 1:**
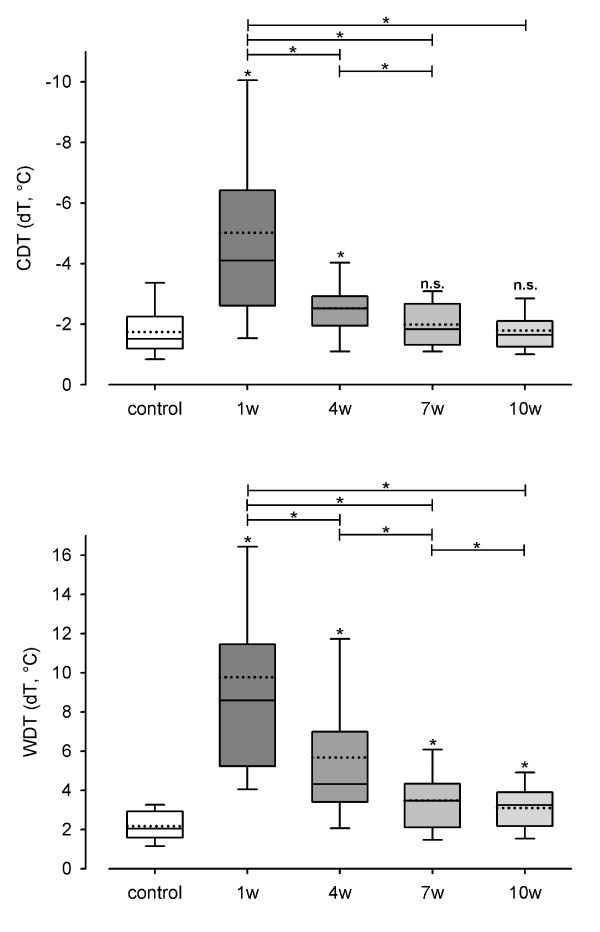
**Monitoring of sensory thresholds in 30 patients and 30 volunteers after oral and maxillofacial surgery**. Cold detection threshold (CDT) and warm detection threshold (WDT) were determined from 120 QST experiments in 30 patients and 30 QST experiments in 30 control subjects. CDT and WDT are given as differences from baseline (32°C; dT). White bars show data of the control group and grey bars (1 w: one week, 4 w: 4 weeks, 7 w: 7 weeks, 10 w: 10 weeks after surgery) present data of test areas. Data on control group and test areas are presented as box plots. Solid lines indicate median, dashed lines the arithmetic mean. Significant differences compared to the control group are indicated by asterisks over the bars (*: p < 0.05; Friedman Repeated Measures ANOVA and subsequent Student-Newman-Keuls test).

**Figure 2:**
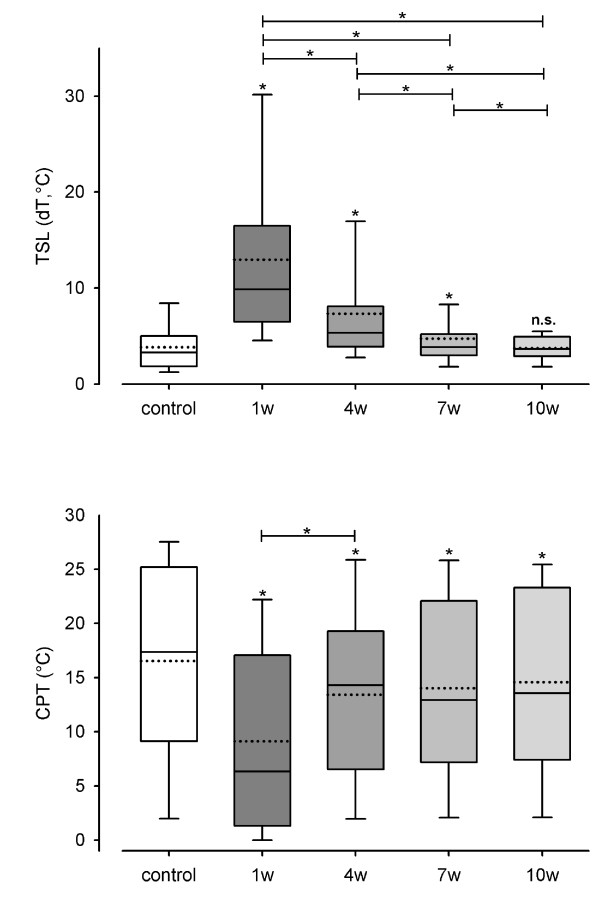
**Monitoring of sensory thresholds in 30 patients and 30 volunteers after oral and maxillofacial surgery**. Thermal sensory limen (TSL) and cold pain threshold (CPT) were determined from 120 QST experiments in 30 patients and 30 QST experiments in 30 control subjects. TSL shows mean differences between temperatures causing warm and cold perceptions. CPT is defined as absolute temperatures (°C). White bars show data of the control group and grey bars (1 w: one week, 4 w: 4 weeks, 7 w: 7 weeks, 10 w: 10 weeks after surgery) present data of test areas. Data on control group and test areas are presented as box plots. Solid lines indicate median, dashed lines the arithmetic mean. Significant differences compared to the control group are indicated by asterisks over the bars (*: p < 0.05; Friedman Repeated Measures ANOVA and subsequent Student-Newman-Keuls test).

**Figure 3:**
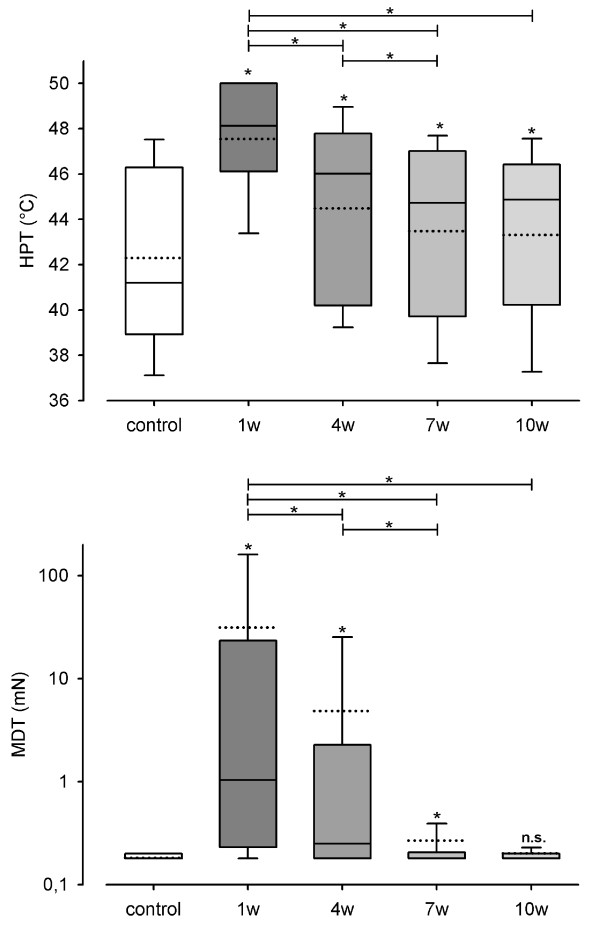
**Monitoring of sensory thresholds in 30 patients and 30 volunteers after oral and maxillofacial surgery**. Heat pain threshold (HPT) and mechanical detection threshold (MDT) were determined from 120 QST experiments in 30 patients and 30 QST experiments in 30 control subjects. HPT is defined as absolute temperatures (°C). MDT values are shown in logarithmic scales. White bars show data of the control group and grey bars (1 w: one week, 4 w: 4 weeks, 7 w: 7 weeks, 10 w: 10 weeks after surgery) present data of test areas. Data on control group and test areas are presented as box plots. Solid lines indicate median, dashed lines the arithmetic mean. Significant differences compared to the control group are indicated by asterisks over the bars (*: p < 0.05; Friedman Repeated Measures ANOVA and subsequent Student-Newman-Keuls test).

**Figure 4:**
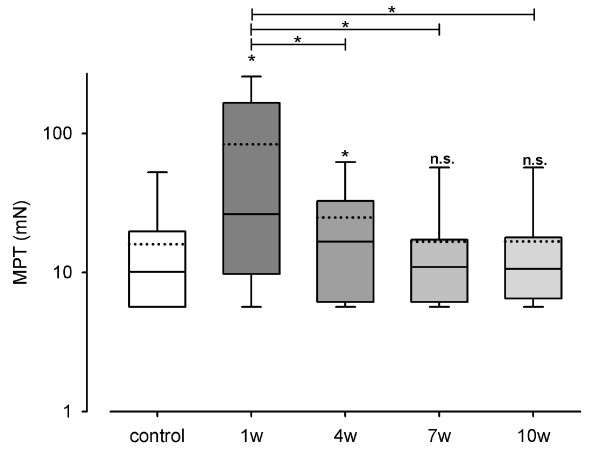
**Monitoring of sensory thresholds in 30 patients and 30 volunteers after oral and maxillofacial surgery**. Mechanical pain threshold (MPT) was determined from 120 QST experiments in 30 patients and 30 QST experiments in 30 control subjects. MPT values are shown in logarithmic scales. White bars show data of the control group and grey bars (1 w: one week, 4 w: 4 weeks, 7 w: 7 weeks, 10 w: 10 weeks after surgery) present data of test areas. Data on control group and test areas are presented as box plots. Solid lines indicate median, dashed lines the arithmetic mean. Significant differences compared to the control group are indicated by asterisks over the bars (*: p < 0.05; Friedman Repeated Measures ANOVA and subsequent Student-Newman-Keuls test).

Significant differences (p < 0.05) between control group and test side 1 week after surgery were shown for CDT (χ^2 ^= 48.530, p < 0.001), WDT (χ^2 ^= 89.310, p < 0.001) (Figure 1), TSL (χ^2 ^= 67.097, p < 0.001), CPT (χ^2 ^= 24.144, p < 0.001) (Figure 2), HPT (χ^2 ^= 36.808, p < 0.001), MDT (χ^2 ^= 76.096, p < 0.001) (Figure 3) and MPT (χ^2 ^= 21.222, p < 0.001) (Figure 4). No significant differences between the median values of the measurements were shown for PHS, MPS, ALL, WUR and VDT (Table 1).

**Table 1:** Mean values ± SD of all QST-parameters in 30 healthy volunteers (control group) and 30 patients.

	*Control group*		*Test* *1 week* *postop*		*Test* *4 weeks* *postop*		*Test* *7 weeks* *postop*		*Test* *10 weeks* *postop*	
CDT (°C)	-1.8	± 1.5	**-5.0**	**± 3.5**	**-2.5**	**± 1.0**	-2.0	± 0.8	-1.8	± 0.7
WDT (°C)	2.4	± 1.8	**9.8**	**± 9.0**	**5.7**	**± 3.6**	**3.5**	**± 1.6**	**3.1**	**± 1.2**
TSL (°C)	3.8	± 2.7	**12.9**	**± 10.8**	**7.3**	**± 6.1**	**4.7**	**± 3.8**	3.7	± 1.4
PHS (x/3)	0.0	± 0.0	0.0	± 0.0	0.0	± 0.0	0.0	± 0.0	0.0	± 0.0
CPT (°C)	16.5	± 9.3	**9.1**	**± 7.8**	**13.4**	**± 8.0**	**14.0**	**± 8.0**	**14.6**	**± 3.7**
HPT (°C)	42.3	± 3.8	**47.5**	**± 2.8**	**44.5**	**± 4.1**	**43.5**	**± 4.1**	**43.3**	**± 3.8**
MDT(mN)	0.2	± 0.0	**31.4**	**± 70.0**	**4.9**	**± 11.1**	**0.3**	**± 0.3**	0.2	± 0.1
MPT(mN)	12.3	± 9.0	**83.4**	**± 99.6**	**24.8**	**± 27.2**	**16.6**	± 16.0	16.8	16.6
MPS	2.4	± 1.9	1.1	± 0.9	1.3	± 0.9	1.4	± 0.9	1.8	± 1.7
ALL	0.0	± 0.0	0.1	± 0.3	0.1	± 0.3	0.1	± 0.3	0.1	± 0.3
WUR	2.4	± 1.5	1.8	± 1.3	2.0	± 1.0	1.9	± 0.7	1.9	± 0.8
VDT (x/8)	7.3	± 0.5	6.9	± 0.7	7.0	± 0.5	6.9	± 0.6	7.0	± 0.5

Bold values indicate significant differences between test sides and control group.

### Differences between control data and test data 4 weeks, 7 weeks and 10 weeks after surgery

CDT on the test side still differed significantly from the control group 4 weeks after surgery, but there were no significant differences between control group and test side in week 7 and week 10.

WDT improved as well, but the differences between the test side and the control group were significant throughout the period of examination. Within 7 weeks, values within the normal range were achieved (WDT after 7 weeks: 3.47°C).

TSL and MDT test side values differed from the control group values 4 and 7 weeks after surgery. There were no differences 10 weeks after surgery.

CPT, HPT test side values did not achieve the level of the control group within the period of examination. Significant differences were persistent up to and inclusively week 10.

After the first two QST investigations 4 weeks after surgery, no more significant differences between control and test side were shown for MPT.

In conclusion, CDT and MPT values converged to the values of the control group the fastest, followed by MDT and TSL. WDT, CPT and HPT test side values still differed significantly from the control group values 10 weeks after surgery, whereas values in normal range were achieved.

### Differences among test side values

MPT decreased only within the first 4 weeks. This is shown by the significant difference between the test side values 1 week after surgery and those 4 weeks after surgery. As the level of the control side values was already achieved then, no further decrease was detected. There were neither significant differences between the MPT test side values of week 4 and week 7, nor between week 7 and week 10.

CDT, HPT and MDT decreased steadily from one investigation to the next, which is shown by the fact that the test side values 1 week after surgery differed significantly from the test side values 4 weeks after surgery and those of week 4 differed significantly from those of week 7, respectively. There were no significant differences between the test side values 7 weeks after surgery and those 10 weeks after surgery, as the level of the control side values was achieved.

WDT also decreased from one investigation to the next. WDT test side values 1 week after surgery differed significantly from those 4 weeks after surgery, and those 4 weeks after surgery differed from WDT test side values 7 weeks after surgery. WDT test side values 7 weeks after surgery differed significantly from those 10 weeks after surgery.

TSL decreased steadily from one investigation to the next, which is shown by the fact that the test side values 1 week after surgery differed significantly from the test side values 4 weeks after surgery and those of week 4 differed significantly from those of week 7. There were significant differences between the test side values 7 weeks after surgery and those 10 weeks after surgery, too.

CPT, however, decreased only from the first investigation to the second. The values of week 1 differed significantly from those of week 4. Then, the decrease stopped. There were neither significant differences between week 4 and week 7, nor between week 7 and week 10.

### Correlations between QST parameters and age

**Figure 5:**
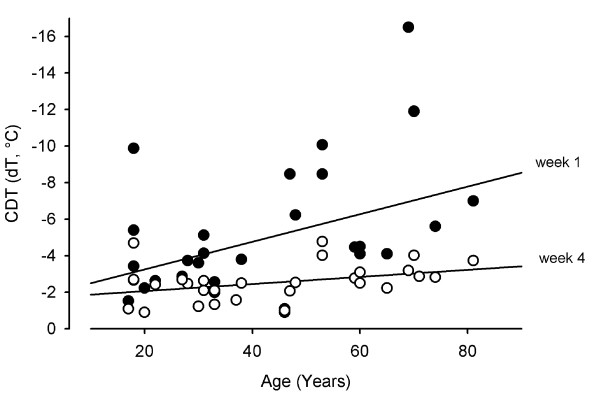
**Correlation between Age and cold detection threshold (CDT) in 30 patients**. CDT is given as difference from baseline (32°C; dT). Data were analyzed by Pearson's correlation analysis. Each point represents the results of one subject on week one (black filled spheres) or on week 4 (white filled spheres). The lines show linear regression curves (upper line: 1 week after surgery, lower line: 4 weeks after surgery).

On week 1 and 4, there was a positive correlation between CDT and age (Figure 5).

## Discussion

The applied QST battery was introduced as a reliable method to investigate sensory function and was recommended as "gold standard" by the German Research Network on Neuropathic Pain (DFNS) [6], but patient data in the perioral region were still missing.

In a literature review, however, it is stated that sensory function was still not uniformly tested and presented, making a comparison of data impossible and highlighting the need for uniform testing methodology [24]. Therefore, normative QST data in clinically relevant perioral regions (extraoral and intraoral) were collected and effects of age, gender, and anatomical sites on QST parameters were analyzed [22]. The present study is based on this study and extends it with data, which have been collected from patients after oral and maxillofacial surgery.

A previous study said that reproducibility was better with only one examiner involved [18] and another study showed poor reproducibility of thermal perception thresholds [25], which may very well be related to the larger number of investigators involved [16]. Therefore, in the present study only one person was instated to do all the measurements.

Inclusion of a control group was recommended by another QST-study [26]. The present study shows that control side values may did not differ from the control group values.

The measurements were finished 10 weeks after surgery, because the most results did not show differences between the test side values 7 and 10 weeks after surgery, and the level of normative data was achieved.

As most nerves with axonal injury show incomplete sensory recovery 1 year after surgery [27], it is assumed that in the present study no axonal injury, but pure demyelinating injuries have occurred. Complete recovery of pure demyelinating injuries after 2 to 4 months corresponds to literature [27,28]. Another explanation for temporary impairment of nerve function could be postoperative injury. A study of a rabbit model showed that functional changes induced by compression are likely due to intraneural edema, which could subsequently result in impairment of nerve function [29].

CDT and MPT reflecting the function in small myelinated Aδ fibres [20,30-32] converged to the values of the control group the fastest, followed by TSL and MDT. MDT reflects myelinated Aβ fibres [30]. WDT reflecting the function in C fibres [33], still differed significantly from the control group values 10 weeks after surgery, whereas values in normal range were achieved, though.

These findings do not correlate with a study, in which the improvement of function in small unmyelinated nerve fibres came within 6 weeks, but the improvement of function in small myelinated fibres was not found before 12 months after surgery [34]. Previous QST-studies, in contrast to the present study, considered the Light Touch Detection Threshold as the most sensitive and most useful test in the follow-up of recovery [24,35]. These different results may be due to the different testing areas and testing methods, making comparisons impossible and underlining the need of a uniform testing method.

Yekta et al. showed an age dependency of quantitative sensory parameters in healthy probands, which demonstrated impairment of sensory function with increasing age [22]. The present study found that older patients tend to be less sensitive than younger patients also in the postoperative stadium.

The testing protocol with 13 parameters has already been considered as too extensive by other studies [36,37]. The present study indicates that 7 of 13 parameters (CDT, MPT, TSL, WDT, CPT, HPT and MDT) are necessary to examine sensory function after oral- and maxillofacial surgery. The development of these parameters would take about 1/2 hour.

Experimental studies of the effects of compression on the pig cauda equine have shown that the recovery of nerve function depends on the magnitude and duration of compression [38,39], but may depend on many more various factors like nerve fibre size [34], grade of injury and surgical technique [37]. To gain better information on intraoperative risk factors, postoperative complications and sensory recovery, a uniform testing method is needed. For this reason, the implementation of QST should be realized at least at university centers and dental clinics. For the measurement of thermal parameters, the acquisition of a computer controlled thermode is required, and for the measurement of MPT and MDT merely a set of pinprick stimulators and Von Frey filaments is needed.

At RWTH Aachen University, QST is already used as an approved instrument to give a neutral expert's opinion in trials.

## Conclusion

In conclusion, somatosensory nerve fiber functions can be assessed in extraoral and intraoral sites by QST. The presented study facilitates the role of QST in diagnosis and monitoring of orofacial nerve fiber dysfunctions. It uses QST in extraoral and intraoral regions following different interventions in oral and maxillofacial surgery. As this QST battery takes 1 hour of testing, it is too time-consuming to realize integration into clinical practice. This study shows that the extent of the testing battery can be reduced to 7 parameters, without affecting the informative value of the measurement.

## Competing interests

The authors declare that they have no competing interests.

## Authors' contributions

SSY conceived of the study, organized and investigated the orofacial sensory function. FK contributed editorial input. MBG, MEO, JS, AG, DR, FL participated in the study design, supported by scientific consulting and coordination and helped to draft the manuscript. RS recruited the patient, organized the study approval by the ethic committee and superviyed the study. All authors read and approved the final manuscript.

## Acknowledgements

Thanks to Lotte Mond and to the laboratory staff.
